# Association of Hormonal Activity with CT-Derived Body Composition Characteristics in Patients with Adrenal Adenomas

**DOI:** 10.3390/jcm15176861

**Published:** 2026-09-04

**Authors:** Yusuf Öztürk, Abdullah Enes Ataş, Şeyma Ünüvar, Muhammet Kocabaş

**Affiliations:** 1 Department of Endocrinology and Metabolism, Sakarya University Training and Research Hospital, Sakarya 54100, Türkiye; 2 Department of Radiology, Faculty of Medicine, Necmettin Erbakan University, Konya 42080, Türkiye; aenesatas@gmail.com; 3 Department of Radiology, Konya City Hospital, Konya 42020, Türkiye; seymababaoglu@hotmail.com; 4 Department of Endocrinology and Metabolism, Faculty of Medicine, Necmettin Erbakan University, Konya 42080, Türkiye; mkocabas@erbakan.edu.tr

**Keywords:** adrenal adenoma, body composition, computed tomography, mild autonomous cortisol secretion, muscle attenuation, skeletal muscle index

## Abstract

**Background/Objectives**: To compare computed tomography (CT)-derived body composition parameters across hormonal subtypes of adrenal adenomas and investigate their associations with hormonal activity. **Methods**: This retrospective study included 171 patients with unilateral adrenal adenomas categorized as Cushing syndrome (CS), mild autonomous cortisol secretion (MACS), non-functioning adenoma (NFA), or primary aldosteronism (PA). Adipose tissue and skeletal muscle parameters were quantified on CT images at the third lumbar vertebral (L3) level. Comparisons were adjusted for age, sex, and body mass index, and associations between hormonal and body composition parameters were assessed. **Results**: After adjustment, significant differences in non-psoas skeletal muscle index (SMI) were observed among the groups. Non-psoas SMI was lower in the CS group than in all other groups and in the MACS group than in the NFA and PA groups. Muscle attenuation was significantly reduced only in the CS group. In multivariable analyses adjusted for age, sex, and BMI, higher post-dexamethasone cortisol levels were independently associated with lower skeletal muscle parameters, with the strongest association observed for non-psoas SMI (β = −0.396, *p* < 0.001). Adipose tissue parameters did not differ significantly among hormonal subtypes. In exploratory analyses, aldosterone levels and the aldosterone-to-renin ratio were positively associated with the visceral-to-subcutaneous fat area ratio. **Conclusions**: Cortisol excess in adrenal adenomas was associated primarily with alterations in skeletal muscle mass and quality, particularly within the non-psoas compartment. Adipose tissue parameters demonstrated more heterogeneous associations with hormonal activity. These findings support further investigation of CT-derived body composition parameters across hormonal subtypes of adrenal adenomas.

## 1. Introduction

Adrenal adenomas are increasingly detected due to the widespread use of imaging modalities [1] and are generally classified as functioning or non-functioning lesions [2,3,4]. Functioning adenomas include cortisol-secreting disorders, such as Cushing syndrome (CS) and the condition currently referred to as Mild Autonomous Cortisol Secretion (MACS), as well as aldosterone-secreting primary aldosteronism (PA) [4,5,6]. Although these subtypes differ substantially in their clinical and metabolic characteristics, their impact on body composition has not been fully elucidated.

Computed tomography (CT)-based measurements at the level of the third lumbar vertebra (L3) are considered a reliable method for quantitative assessment of body composition [7,8,9]. Using a single CT slice at this level, adipose tissue can be quantified as visceral and subcutaneous compartments, whereas skeletal muscle can be assessed through parameters such as total skeletal muscle area and psoas muscle area [10,11]. Although several studies have investigated body composition in patients with adrenal adenomas, most have focused on specific hormonal conditions, particularly overt hypercortisolism or autonomous cortisol secretion, and comparative data encompassing the full spectrum of hormonally distinct adrenal adenomas remain limited [12,13,14,15]. In a large cross-sectional study, hormonal activity was shown to be associated with differences in body composition among patients with adrenal tumors [8]. However, that study included a heterogeneous population with varying pathological and hormonal characteristics and was not specifically focused on adrenal adenomas. Furthermore, the inclusion of bilateral adrenal lesions may complicate the interpretation of lesion-specific hormonal activity, as hormonal assessment is performed at the patient level and the contribution of each individual adrenal lesion cannot always be determined. Therefore, studies evaluating unilateral adrenal adenomas and examining the relationship between CT-derived body composition parameters and hormonal profiles are warranted. In addition, whether different skeletal muscle compartments demonstrate distinct associations with hormonal activity has not been adequately explored.

Accordingly, this study aimed to compare CT-derived body composition parameters measured at the L3 level across hormonal subtypes of adrenal adenomas and to investigate their associations with hormonal activity. In addition to conventional skeletal muscle measurements, psoas and non-psoas muscle compartments were evaluated separately to explore potential differences in their relationship with hormonal status.

## 2. Materials and Methods

### 2.1. Study Design and Patient Selection

This single-center retrospective study was conducted through a collaboration between the Departments of Radiology and Endocrinology and Metabolism. Patients aged ≥18 years with an adrenal mass who underwent complete hormonal evaluation between 1 January 2020 and 31 December 2024 were eligible for inclusion. The study was approved by the local Institutional Review Board (approval date: 24 January 2025; approval number: 2025/5475). Individual informed consent was not obtained for this retrospective study based on retrospectively collected clinical data.

Patients were excluded if they had non-adenomatous adrenal lesions (including adrenal hyperplasia, adrenal thickening, and other non-adenomatous lesions), tumors with hormonal co-secretion, bilateral adrenal lesions, malignant adrenal tumors, pheochromocytoma, adrenocorticotropic hormone (ACTH)-dependent hypercortisolism, a history of extra-adrenal malignancy, systemic glucocorticoid use, CT images unsuitable for body composition analysis, or incomplete clinical or biochemical data. Hormonal co-secretion was defined as the concomitant presence of autonomous cortisol secretion and primary aldosteronism in the same patient.

**Figure 1:**
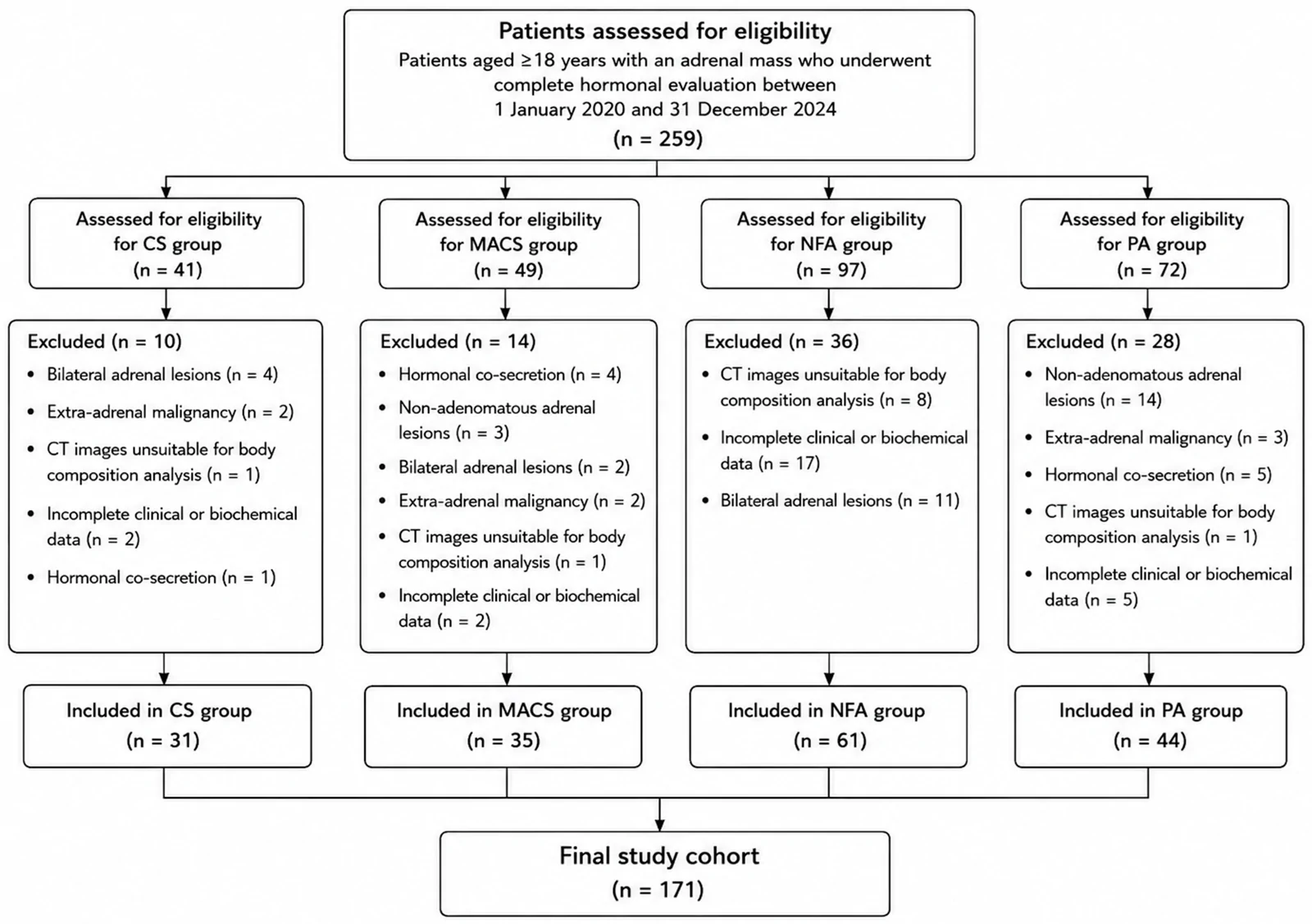
Flow diagram of patient selection.

An adrenal nodule was defined as a discrete focal lesion distinguishable from the surrounding adrenal parenchyma, whereas adrenal enlargement or thickening without a discrete focal lesion was considered adrenal hyperplasia or adrenal thickening. The diagnosis of adrenal adenoma was established based on imaging and/or histopathological characteristics. Lesions with an attenuation value ≤10 HU on unenhanced CT were considered consistent with adrenal adenoma. For lesions with an attenuation value >10 HU on unenhanced CT, additional characterization was performed using adrenal MRI, with signal loss on out-of-phase chemical-shift imaging considered consistent with an adrenal adenoma; when available, histopathological examination was also used for confirmation. Lesions that could not be confirmed as adrenal adenomas according to these criteria were excluded from the study. The patient selection process and reasons for exclusion are summarized in Figure 1.

### 2.2. Hormonal Classification

Patients were categorized into four groups according to hormonal status: CS, MACS, NFA, PA.

CS was diagnosed in patients with clinical features of overt hypercortisolism, including characteristic Cushingoid features such as facial plethora, wide purple striae, easy bruising, proximal muscle weakness, and dorsocervical or supraclavicular fat accumulation, together with biochemical evidence of autonomous cortisol secretion. In addition to cortisol nonsuppression following the 1 mg DST, hypercortisolism was confirmed by at least one additional biochemical assessment, including elevated 24 h urinary free cortisol and/or elevated midnight serum cortisol levels. Plasma ACTH levels were suppressed or low in the great majority of patients, supporting ACTH-independent cortisol secretion. Late-night salivary cortisol was not assessed because this test was not available at our institution during the study period.

MACS was defined as a post-DST serum cortisol level >1.8 µg/dL in the absence of clinical features of overt Cushing syndrome. Abnormal DST results were confirmed by repeat testing on at least one additional occasion, after consideration of potential factors that could lead to false-positive DST results. Morning plasma ACTH and DHEAS levels were used as part of the biochemical assessment of ACTH-independent cortisol secretion. A morning ACTH concentration <10 ng/L was considered suppressed. Of the 35 patients with MACS, 15 had ACTH concentrations <10 ng/L, whereas the remaining 20 patients had low DHEAS levels as a supportive biochemical finding of ACTH-independent cortisol secretion. DHEAS was measured in all patients with MACS. Twenty-four-hour urinary free cortisol was additionally assessed when clinically indicated.

NFA was defined by the absence of biochemical evidence of adrenal hormonal hyperfunction. Cortisol excess was excluded by a serum cortisol level ≤1.8 µg/dL following the 1 mg DST, while PA was excluded based on plasma aldosterone concentration, plasma renin activity (PRA), and the corresponding aldosterone-to-renin ratio (ARR). In 12 patients with initially equivocal or suspicious renin–aldosterone screening results, the saline infusion test (SIT) was performed, and PA was excluded based on the confirmatory test results. Other functional adrenal disorders were also excluded as part of the routine hormonal evaluation.

PA screening was based on plasma aldosterone concentration and PRA, with calculation of the ARR. Blood samples for PA screening were obtained in the seated position under standardized conditions, with correction of hypokalemia before testing and appropriate management of antihypertensive medications known to interfere with the renin–aldosterone axis. An ARR > 20 ([ng/dL]/[ng/mL/h]) together with a plasma aldosterone concentration >10 ng/dL was considered a positive screening result. Confirmatory testing with the SIT was performed in 35 of the 44 patients with PA, with the test conducted in the supine position. A post-SIT aldosterone concentration >10 ng/dL was considered confirmatory for PA. In five patients with post-SIT aldosterone concentrations within the indeterminate range of 6–10 ng/dL, the diagnosis was confirmed by additional biochemical evaluation, including the captopril challenge test. In patients with spontaneous (non-diuretic-induced) hypokalemia, a basal plasma aldosterone concentration >20 ng/dL, and suppressed renin (PRA < 0.6 ng/mL/h or direct renin concentration below the assay detection limit), PA was diagnosed without additional confirmatory suppression testing. For assessment of PA lateralization, adrenal vein sampling (AVS) was performed in 29 of the 44 patients with PA, of whom 21 subsequently underwent adrenalectomy. In 19 of these patients, the decision to proceed with adrenalectomy was based on AVS evidence of unilateral disease. In selected young patients (<35 years) with marked hypokalemia and a clear unilateral adrenal adenoma >1 cm on CT, AVS was not required when the clinical, biochemical, and imaging findings were concordant with unilateral disease. Fifteen patients meeting these criteria underwent adrenalectomy without AVS. Overall, 36 patients with PA underwent adrenalectomy, and the diagnosis of adrenal adenoma was confirmed histopathologically.

### 2.3. Clinical and Biochemical Assessment

Demographic characteristics, comorbidities, and laboratory data were retrospectively obtained from electronic medical records. Clinical parameters, including age, sex, body mass index (BMI), and waist circumference, were recorded.

Hypertension was defined as a previous diagnosis of hypertension or the use of at least one antihypertensive medication. Diabetes mellitus was defined as a pre-existing diagnosis or the use of antidiabetic therapy.

All biochemical and hormonal analyses were performed in the central laboratory between 1 January 2020 and 31 December 2024 as part of routine clinical practice using standardized methods. Serum glucose, glycated hemoglobin (HbA1c), lipid profile, creatinine, sodium, and potassium levels were measured using automated biochemical analyzers. Serum cortisol, adrenocorticotropic hormone (ACTH), and dehydroepiandrosterone sulfate (DHEAS) were measured using electrochemiluminescence immunoassays (ECLIA; Roche Diagnostics, Mannheim, Germany). Competitive ECLIA was used for cortisol and DHEAS, whereas a sandwich ECLIA was used for ACTH. The reference intervals were 6.02–18.4 µg/dL in the morning and 2.68–10.5 µg/dL in the afternoon for cortisol, 7.2–63.3 ng/L for ACTH, and 19–407 µg/dL for DHEAS. Plasma aldosterone concentration was measured using a competitive enzyme-linked immunosorbent assay (ELISA; DRG Diagnostics, Marburg, Germany). Aldosterone concentrations were converted to ng/dL for the present analyses, with a corresponding reference interval of 1.46–17.4 ng/dL. PRA was measured using an immunoassay (DRG Diagnostics, Marburg, Germany), with reference intervals of 0.295–3.2 ng/mL/h in the upright position and 0.051–2.69 ng/mL/h in the supine position.

The 1 mg DST was performed by administering 1 mg dexamethasone at 11:00 p.m., followed by measurement of serum cortisol levels the following morning.

### 2.4. CT Imaging and Body Composition Analysis

Abdominal computed tomography (CT) images of all patients were retrospectively reviewed. CT examinations were performed between 1 January 2020 and 31 December 2024 at the same institution using a 256-slice dual-energy CT scanner (SOMATOM Drive; Siemens Healthineers, Forchheim, Germany) according to the institution’s standardized adrenal CT protocol. The same standardized imaging protocol was used for all patients throughout the study period. All images were retrieved from the institutional archive in Digital Imaging and Communications in Medicine (DICOM) format.

Body composition analysis, including muscle attenuation measurements, was performed exclusively on axial unenhanced CT images obtained at the level of the third lumbar vertebra (L3). This level was selected because of its strong correlation with whole-body skeletal muscle and adipose tissue mass. Segmentation and image analysis were performed using 3D Slicer software (version 5.6). Tissue segmentation was performed manually. Hounsfield unit (HU) thresholds of −190 to −30 HU for adipose tissue and −29 to +150 HU for skeletal muscle were applied.

CT imaging and hormonal evaluation were performed as part of the same diagnostic work-up, with an interval of no more than one month between the two assessments in all patients. In patients evaluated because of clinical suspicion of hormonal activity, adrenal CT was performed following hormonal assessment, whereas in patients with incidentally detected adrenal lesions, hormonal evaluation was completed after CT imaging. All CT examinations used for body composition analysis were obtained before adrenal surgery or the initiation of specific treatment for hormonal excess.

At the L3 level, visceral fat area (VFA, cm^2^), subcutaneous fat area (SFA, cm^2^), psoas muscle area (cm^2^), non-psoas skeletal muscle area (cm^2^), and total skeletal muscle area (cm^2^) were measured. Total skeletal muscle area included the psoas, paraspinal, quadratus lumborum, transversus abdominis, internal and external oblique, and rectus abdominis muscles. Non-psoas skeletal muscle area was calculated by subtracting the bilateral psoas muscle area from the total skeletal muscle area. Muscle attenuation was defined as the mean HU value of the skeletal muscle area.

Muscle areas were normalized for height squared and expressed as the psoas muscle index (PMI, cm^2^/m^2^), non-psoas skeletal muscle index (non-psoas SMI, cm^2^/m^2^), and total skeletal muscle index (SMI, cm^2^/m^2^), consistent with established approaches used in CT-based skeletal muscle assessment [7,10]. In addition, the VFA-to-SFA ratio was calculated to assess abdominal fat distribution.

All measurements were independently performed by two experienced radiologists who were blinded to the clinical and hormonal diagnoses. Interobserver agreement was assessed using the intraclass correlation coefficient (ICC). Excellent interobserver agreement was observed for the evaluated imaging measurements, with individual ICC values and 95% confidence intervals presented in Supplementary Table S1. Given the excellent interobserver agreement, measurements obtained by the primary reader were used for statistical analyses.

### 2.5. Statistical Analysis

Statistical analyses were performed using IBM SPSS Statistics software (version 22.0; IBM Corp., Armonk, NY, USA). The distribution of continuous variables was assessed using the Kolmogorov–Smirnov test. Normally distributed variables are presented as mean ± standard deviation, whereas non-normally distributed variables are presented as median (interquartile range). Categorical variables are expressed as frequencies and percentages.

For comparisons among groups, one-way analysis of variance (ANOVA) was used for normally distributed continuous variables, whereas the Kruskal–Wallis test was used for non-normally distributed variables. Pairwise comparisons were performed using Bonferroni correction when a significant overall difference was detected.

Analysis of covariance (ANCOVA) was used to adjust for the potential effects of age, sex, and BMI. Associations between hormonal parameters and body composition variables were evaluated using Spearman correlation analysis. To further assess the associations between post-DST cortisol levels and skeletal muscle parameters, multivariable linear regression analyses were performed in patients with CS, MACS, and NFA. In Model 1, analyses were adjusted for age, sex, and BMI. Model 2 additionally included hormonal subgroup (CS, MACS, or NFA) to evaluate whether the associations with post-DST cortisol persisted after accounting for diagnostic category. Standardized regression coefficients (β) and *p* values were reported. Patients with PA were excluded from these analyses. Interobserver agreement was assessed using the ICC, based on a two-way random-effects model for absolute agreement using single measurements. ICC values were reported with 95% confidence intervals (CIs).

A two-sided *p* value < 0.05 was considered statistically significant.

## 3. Results

A total of 171 patients with adrenal adenomas were included in the study and classified according to hormonal subtype as CS (*n* = 31), MACS (*n* = 35), NFA (*n* = 61), and PA (*n* = 44). Adrenalectomy was performed in 76 patients, including 31 patients with CS, 5 with MACS, 4 with NFA, and 36 with PA. Demographic, clinical, biochemical, and hormonal characteristics are summarized in Table 1. Significant differences in age were observed among the groups, with the highest age recorded in the MACS group (*p* < 0.001). Sex distribution also differed significantly among the groups, with the highest proportion of female patients observed in the CS group (*p* = 0.019). No significant differences were found in body mass index or waist circumference (*p* = 0.059 and *p* = 0.995, respectively). The prevalence of hypertension differed significantly among the groups, and all patients in the PA group had hypertension (*p* < 0.001). No significant differences were observed in the prevalence of diabetes mellitus or prediabetes (*p* = 0.122 and *p* = 0.331, respectively). Among the biochemical parameters, HbA1c levels were significantly higher in the CS group (*p* = 0.013). Total cholesterol levels also differed significantly among the groups, with the highest values observed in the CS group (*p* = 0.028).

Unadjusted CT-derived body composition parameters are presented in Supplementary Table S2. In the unadjusted analyses, significant differences were observed among the groups in tumor size and several skeletal muscle parameters, whereas VFA, SFA, and the VFA-to-SFA ratio did not differ significantly.

Age-, sex-, and body mass index-adjusted CT-derived body composition parameters are presented in Table 2, and pairwise comparisons are shown in Table 3. No significant differences were observed in VFA or SFA after adjustment (*p* = 0.133 and *p* = 0.072, respectively). A significant overall difference was observed in the VFA-to-SFA ratio (*p* = 0.039); however, none of the individual pairwise comparisons remained statistically significant after Bonferroni correction. No significant differences were found in psoas muscle area or PMI (*p* = 0.221 and *p* = 0.247, respectively), and pairwise comparisons revealed no significant differences between any groups. Significant differences were observed in non-psoas SMI (CS–MACS, *p* = 0.017; CS–NFA, *p* < 0.001; CS–PA, *p* < 0.001; MACS–NFA, *p* = 0.017; and MACS–PA, *p* = 0.002). Significant differences were also observed in total SMI (CS–MACS, *p* = 0.009; CS–NFA, *p* < 0.001; CS–PA, *p* < 0.001; and MACS–PA, *p* = 0.013). Muscle attenuation differed significantly among the groups, with significant differences observed between the CS group and all other groups (CS–MACS, *p* = 0.025; CS–NFA, *p* = 0.001; and CS–PA, *p* < 0.001).

Correlations between hormonal parameters and CT-derived body composition variables are presented in Table 4. Cortisol levels assessed by the 1 mg dexamethasone suppression test (DST) were significantly correlated with psoas muscle area (r = −0.237, *p* = 0.011), non-psoas skeletal muscle area (r = −0.554, *p* < 0.001), total skeletal muscle area (r = −0.519, *p* < 0.001), PMI (r = −0.229, *p* = 0.014), non-psoas SMI (r = −0.496, *p* < 0.001), total SMI (r = −0.484, *p* < 0.001), and muscle attenuation (r = −0.384, *p* < 0.001). No significant correlations were observed between DST cortisol levels and VFA, SFA, or the VFA-to-SFA ratio (all *p* > 0.05). Significant positive correlations were identified between aldosterone levels and the VFA-to-SFA ratio (r = 0.308, *p* = 0.009), as well as between ARR and the VFA-to-SFA ratio (r = 0.272, *p* = 0.021).

To further evaluate the associations between cortisol excess and skeletal muscle parameters, multivariable linear regression analyses were performed in patients with CS, MACS, and NFA (Table 5). After adjustment for age, sex, and BMI (Model 1), higher post-DST cortisol levels remained significantly associated with lower psoas muscle area, non-psoas skeletal muscle area, total skeletal muscle area, PMI, non-psoas SMI, and total SMI, as well as lower muscle attenuation (all *p* < 0.05). After additional adjustment for hormonal subgroup (Model 2), the inverse associations remained significant for non-psoas skeletal muscle area (β = −0.214, *p* = 0.019), total skeletal muscle area (β = −0.205, *p* = 0.041), non-psoas SMI (β = −0.251, *p* = 0.011), total SMI (β = −0.209, *p* = 0.034), and muscle attenuation (β = −0.291, *p* = 0.019), whereas the associations with psoas muscle area and PMI were no longer statistically significant (*p* = 0.310 and *p* = 0.220, respectively).

## 4. Discussion

Although several studies have evaluated the relationship between hormonal activity and body composition in adrenal adenomas, comprehensive investigations of the effects of different hormonal subtypes on CT-derived body composition parameters remain limited. In the present study, we evaluated CT-derived body composition parameters across hormonal subtypes and investigated their associations with hormonal status in a homogeneous cohort of patients with unilateral adrenal adenomas without hormonal co-secretion. In addition to adipose tissue parameters, skeletal muscle composition was analyzed separately in the psoas and non-psoas muscle compartments. Our findings demonstrated significant associations between cortisol excess and skeletal muscle parameters, particularly within the non-psoas muscle compartment. Furthermore, significant correlations were observed between post-dexamethasone cortisol levels and multiple skeletal muscle parameters, with the strongest associations identified for non-psoas muscle mass and non-psoas SMI. Patients with MACS exhibited an intermediate phenotype between those with CS and NFA across most body composition parameters. In contrast, adipose tissue parameters showed fewer differences among hormonal subtypes, although significant associations with parameters reflecting visceral fat distribution were observed in the PA group.

One of the most notable findings of our study was the marked difference in skeletal muscle-related parameters across hormonal subtypes. Lower skeletal muscle mass was observed in adrenal adenomas associated with cortisol excess, and these alterations appeared to be particularly evident within the non-psoas muscle compartment. Previous studies have demonstrated an association between endogenous hypercortisolism and reduced muscle mass or sarcopenia. Delivanis et al. reported that increasing post-DST cortisol levels were associated with lower total abdominal skeletal muscle mass in patients with adrenal adenomas [16]. Similarly, in a large cross-sectional study, patients with CS exhibited lower skeletal muscle area, and post-DST cortisol levels were negatively associated with skeletal muscle parameters [8]. While our findings support these observations, an important aspect of our study is the separate evaluation of psoas and non-psoas muscle compartments. Most previous studies have relied on either total skeletal muscle area or PMI alone. Notably, although PMI was lower in the CS group in unadjusted analyses, this association lost statistical significance after adjustment for age, sex, and BMI. In contrast, non-psoas SMI remained significantly lower in the CS group than in the other groups after adjustment. This pattern was also observed in the additional multivariable analyses: the inverse associations of post-DST cortisol with non-psoas skeletal muscle area and non-psoas SMI remained statistically significant after adjustment for age, sex, BMI, and hormonal subgroup, whereas the corresponding associations with psoas muscle area and PMI were no longer statistically significant. Taken together, these findings raise the possibility that non-psoas measurements may be more sensitive than isolated psoas measurements in capturing skeletal muscle alterations associated with cortisol excess. However, the presence of statistically significant associations for non-psoas measures but not for psoas-specific measures does not, by itself, establish a statistically significant difference in the magnitude of the associations between the two muscle compartments. Furthermore, because the non-psoas compartment represents a substantially larger muscle area than the psoas alone, greater measurement precision and statistical power may have contributed to the observed pattern. Therefore, these findings should be regarded as hypothesis-generating rather than as evidence of preferential biological involvement of the non-psoas musculature, and direct comparisons of muscle compartments in appropriately designed studies are warranted. One possible biological explanation for the observed pattern may relate to differences in muscle composition and susceptibility to glucocorticoid effects. Glucocorticoids are known to preferentially promote protein catabolism in fast-twitch type II muscle fibers, whereas slow-twitch type I fibers appear relatively resistant to these effects [17]. The psoas major muscle contains a substantial proportion of type I fibers and serves both postural and dynamic functions [18]. However, the non-psoas compartment evaluated in our study comprises several anatomically and functionally distinct muscles and cannot be assumed to have a uniform fiber-type composition. Although previous studies have shown that iliopsoas muscle area is reduced in patients with overt CS and may reflect cortisol excess [11], these studies did not directly compare the psoas muscle with total skeletal muscle or other muscle compartments. Thus, although differences in muscle fiber composition may represent one possible explanation for the observed pattern, our CT-based findings cannot directly establish that this pattern is driven by selective glucocorticoid effects on specific muscle fiber types. Further studies incorporating muscle-specific functional, histological, or molecular assessments are needed to clarify the biological mechanisms underlying these findings.

In addition to alterations in skeletal muscle mass, our findings suggest that cortisol excess also has a substantial impact on muscle quality. The observed reduction in muscle attenuation indicates that cortisol excess may influence not only muscle quantity but also muscle quality, potentially reflecting changes in muscle composition, including increased intramuscular fat content. Previous studies have demonstrated associations between autonomous cortisol secretion and lower skeletal muscle density (SMD), a CT-derived marker of muscle quality, as well as increased intermuscular adipose tissue (IMAT). Moreover, these alterations have been reported to improve, at least partially, following adrenalectomy [19]. Similarly, a large cross-sectional study reported lower muscle attenuation in patients with CS [8]. Consistent with these findings, muscle attenuation was significantly lower in the CS group in our study, further supporting an association between cortisol excess and alterations in muscle quality. Furthermore, the significant inverse correlation between post-DST cortisol levels and muscle attenuation suggests that the impact of hypercortisolism on muscle quality may be related to the degree of hormonal activity. In contrast, differences in muscle attenuation were not evident in the MACS group. This finding may suggest that alterations in muscle attenuation are more apparent in overt cortisol excess, although longitudinal studies are required to clarify the temporal relationship between changes in muscle mass and muscle quality.

Another noteworthy finding of our study was that non-psoas SMI remained significantly lower in patients with MACS compared with those with NFA after adjustment for age, sex, and BMI. Although previous studies have demonstrated reductions in total skeletal muscle mass in overt hypercortisolism, muscle alterations associated with milder degrees of cortisol excess have been reported less consistently [16]. Notably, large cross-sectional studies have failed to demonstrate significant differences in total skeletal muscle parameters between patients with MACS and those with NFA, suggesting that conventional muscle measurements may not fully capture muscle alterations associated with mild degrees of cortisol excess [8]. In contrast, non-psoas muscle parameters remained significantly altered in the MACS group in our study, suggesting that muscle alterations associated with mild cortisol excess may be more evident in certain skeletal muscle compartments. Taken together, our findings suggest that skeletal muscle alterations may be present across different degrees of cortisol excess. However, the temporal sequence of changes in muscle mass and muscle quality cannot be determined from the present cross-sectional study. In this context, non-psoas muscle parameters derived from routine abdominal CT examinations may provide additional information regarding body composition changes associated with mild cortisol excess.

Compared with skeletal muscle parameters, differences in adipose tissue parameters across hormonal subtypes were more limited in our study. Although increased visceral adiposity is a well-established feature of overt Cushing syndrome, findings regarding fat distribution in milder forms of cortisol excess have been more heterogeneous. In a large cross-sectional study, patients with CS exhibited significantly greater visceral adiposity, whereas patients with MACS and NFA demonstrated similar body composition profiles [8]. In our study, however, no significant differences in VFA, SFA, or the VFA-to-SFA ratio were observed after adjustment for age, sex, and BMI, not only between the MACS and NFA groups but also when the CS group was included in the comparison. Furthermore, the absence of significant associations between post-DST cortisol levels and adipose tissue parameters suggests that the effects of mild cortisol excess on fat distribution may be limited or heterogeneous. Despite adjustment for BMI, the overall burden of visceral adiposity across all groups may have obscured additional effects attributable to cortisol excess. Moreover, previous studies have reported increased visceral fat accumulation, reduced muscle mass, and elevated cardiometabolic risk even in patients with NFA, suggesting that this group may not represent a metabolically normal reference population [13]. This may partly explain the lack of substantial differences in adipose tissue parameters between the NFA and MACS groups in our cohort. Taken together, these findings suggest that skeletal muscle and adipose tissue parameters may demonstrate different patterns of association with cortisol excess.

In our exploratory correlation analyses, associations were observed between fat distribution and mineralocorticoid activity in the PA group. In particular, the positive correlations of aldosterone levels and ARR with the VFA-to-SFA ratio suggest a potential relationship between mineralocorticoid activity and abdominal fat distribution. Previous studies investigating the relationship between primary aldosteronism and abdominal fat distribution have yielded heterogeneous results. While some studies have reported an association between aldosterone excess and increased visceral adiposity, others have demonstrated lower abdominal fat accumulation, particularly in patients with aldosterone-producing adenomas (APA) [14]. More recently, a large cross-sectional study reported that patients with PA had similar VFA but lower SFA than age-, sex-, and BMI-matched controls, suggesting a relative predominance of visceral adiposity [8]. The positive correlations of aldosterone levels and ARR with the VFA-to-SFA ratio observed in our cohort are also consistent with previous findings suggesting that mineralocorticoid activity may be associated with the relative distribution of visceral and subcutaneous adipose tissue. However, these findings were derived from exploratory correlation analyses involving multiple hormonal and body composition parameters, without adjustment for multiple testing. Therefore, these associations should be interpreted cautiously and require confirmation in future studies.

This study has several limitations. First, the retrospective and cross-sectional design precludes the establishment of causal relationships. In addition, the single-center nature of the study and the relatively small sample sizes of the CS, MACS, and PA subgroups may limit the generalizability of the findings. Although hormonal classification was based on detailed biochemical evaluations and repeated or confirmatory testing when appropriate, the possibility of hormonal misclassification cannot be completely excluded because of the retrospective nature of the study and the biological variability of hormonal measurements. Second, the duration of cortisol and aldosterone excess could not be assessed. Given that the observed alterations in skeletal muscle and adipose tissue may be related to cumulative hormonal exposure, reliance on single-time-point hormonal measurements may not fully reflect the true burden of hormonal activity. In addition, some demographic characteristics, including age and sex, differed across the hormonal subgroups. Although the analyses were adjusted for age, sex, and BMI, residual confounding related to these differences cannot be completely excluded. Furthermore, because hormonal subgroup classification is partly based on post-DST cortisol concentrations, their simultaneous inclusion in multivariable analyses may introduce collinearity, which should be considered when interpreting the findings. Third, body composition was evaluated solely using CT-derived morphologic parameters, and no functional assessments of muscle performance, such as handgrip strength, physical performance, or gait speed, were available. Consequently, the relationship between alterations in muscle mass or muscle quality and functional clinical outcomes could not be evaluated. Furthermore, information regarding physical activity, dietary habits, protein intake, smoking and alcohol use, as well as detailed information on medications that may influence muscle and adipose tissue composition, particularly lipid-lowering and glucose-lowering therapies, was not consistently available. Therefore, residual confounding related to these factors cannot be excluded. Fourth, body composition analysis was performed using a single axial CT slice at the L3 level. Although this approach is widely used and well validated for the assessment of whole-body muscle and adipose tissue composition, it provides less comprehensive information than volumetric analyses. Finally, the present study was not designed to evaluate the diagnostic or prognostic performance of CT-derived body composition parameters, and no longitudinal follow-up data were available to assess their clinical utility over time. In addition, the evaluation of associations between multiple hormonal parameters and body composition variables, including the additional multivariable regression analyses across several muscle outcomes, involves multiple statistical comparisons and may increase the risk of type I error. Therefore, the exploratory correlation analyses and the additional regression findings should be interpreted cautiously and require confirmation in independent cohorts.

Despite these limitations, the study has several important strengths. To our knowledge, this is the first study to evaluate the relationship between hormonal activity and skeletal muscle composition in adrenal adenomas by separately assessing the psoas and non-psoas muscle compartments. Furthermore, the simultaneous evaluation of muscle mass, muscle attenuation, and fat distribution across different hormonal subtypes provides a more comprehensive understanding of body composition alterations in adrenal adenomas. Finally, all imaging analyses were performed independently by two experienced radiologists who were blinded to the hormonal diagnoses, and interobserver agreement was excellent for the evaluated imaging parameters (ICC >0.90), supporting the reliability of the measurements. From a clinical perspective, CT-derived skeletal muscle parameters may provide complementary information regarding the systemic consequences of cortisol excess using imaging already routinely obtained during adrenal evaluation. The stronger associations observed for the non-psoas muscle compartment suggest that this compartment may be particularly sensitive to cortisol-related alterations in skeletal muscle. However, larger prospective studies with longitudinal assessments are needed to determine the clinical significance of these findings and to evaluate their relationship with changes following adrenalectomy.

## 5. Conclusions

In conclusion, cortisol excess in adrenal adenomas was associated primarily with alterations in skeletal muscle mass and muscle quality. Associations with skeletal muscle parameters were more evident in the non-psoas muscle compartment, whereas adipose tissue parameters demonstrated more heterogeneous relationships with hormonal activity. Exploratory analyses also suggested a possible relationship between mineralocorticoid activity and abdominal fat distribution, although this finding requires confirmation in future studies. These findings provide further insight into body composition alterations across different hormonal subtypes of adrenal adenomas and suggest that routinely acquired CT examinations may provide additional information regarding the systemic effects of hormonal activity beyond adrenal lesion characterization alone. Although the present findings do not establish a diagnostic role for CT-derived body composition parameters, future prospective studies with larger cohorts are warranted to determine whether specific body composition parameters and potential threshold values could contribute to the hormonal characterization and risk stratification of adrenal adenomas.

## Figures and Tables

**Table 1:** Demographic, clinical, biochemical, and hormonal characteristics of the study population according to adrenal adenoma subtype.

Variable	CS (*n* = 31)	MACS (*n* = 35)	NFA (*n* = 61)	PA (*n* = 44)	*p*-Value
Age (years)	43 (35–49)	61 (46–65)	52 (43–61)	49.5 (43–61)	<0.001 *
Female sex, *n* (%)	27 (87.1)	18 (51.4)	38 (62.3)	30 (68.2)	0.019 *
BMI (kg/m^2^)	33.2 (31.1–37.0)	32.4 (27.8–36.3)	31.1 (26.4–34.5)	31.2 (28.3–34.4)	0.059
WC (cm)	87 (83–95)	86 (80–93)	87 (83–93)	88.5 (82–92)	0.995
HT, *n* (%)	9 (29.0)	19 (54.3)	24 (39.3)	44 (100)	<0.001 *
DM, *n* (%)	15 (48.4)	13 (37.1)	15 (24.6)	13 (29.5)	0.122
PreDM, *n* (%)	5 (16.1)	6 (17.1)	5 (8.2)	3 (6.8)	0.331
Urea (mg/dL)	28.5 (23.7–34.8)	27 (21–39.1)	27 (22–33.5)	28 (23–36)	0.970
Creatinine (mg/dL)	0.73 (0.64–0.82)	0.82 (0.69–0.91)	0.77 (0.68–0.92)	0.83 (0.70–1.03)	0.047 *
Sodium (mmol/L)	140 (139–142.3)	140 (138–142)	140 (139–142)	142 (141–144)	<0.001 *
Potassium (mmol/L)	4.32 (3.89–4.61)	4.40 (4.10–4.80)	4.47 (4.21–4.68)	3.21 (2.94–4.00)	<0.001 *
Fasting glucose (mg/dL)	110 (85–162)	105 (87.9–136)	99 (87.5–119)	99.5 (86.5–122)	0.522
HbA1c (%)	6.4 (5.5–7.5)	6.25 (5.9–6.75)	6.0 (5.7–7.2)	5.6 (5.3–6.05)	0.013 *
Total cholesterol (mg/dL)	217 (187–249)	172 (165–200)	200.5 (159–227)	179 (154–217)	0.028 *
Triglycerides (mg/dL)	130 (100–208)	162 (126–184)	148.5 (105–207)	129.5 (100–178)	0.174
HDL-C (mg/dL)	52.5 (47–65)	40 (34–46)	40.5 (36–49)	45 (40–50)	<0.001 *
LDL-C (mg/dL)	135.5 (99–159)	111 (92–124)	114.5 (81–144)	106 (91–135)	0.253
ACTH (ng/L)	2.08 (1.5–7.94)	12.7 (9.35–17.25)	20.8 (11.1–27.9)	22 (12.9–35.5)	<0.001 *
Serum cortisol (morning, µg/dL)	17.3 (15.1–23.8)	14.1 (9.0–17.0)	11.4 (7.9–16.1)	11.2 (8.35–13.2)	<0.001 *
DHEAS (µg/dL)	38.4 (22.1–72.4)	34 (18.4–62)	82 (38.5–145.5)	69 (52–182)	<0.001 *
1 mg DST cortisol (µg/dL)	16.1 (5.38–19)	3.44 (3.08–6.12)	1.20 (0.85–1.62)	1.28 (0.92–1.51)	<0.001 *
Renin (ng/mL/h)	2.19 (1.75–2.46)	0.95 (0.71–1.28)	1.5 (1.0–2.68)	0.55 (0.21–1.12)	<0.001 *
Aldosterone (ng/dL)	16.8 (12.8–26.0)	8.95 (4.35–20)	16 (8–28)	28 (17.6–45)	<0.001 *
ARR	8.50 (5.62–10.92)	9.72 (4.20–13.13)	8.67 (5.97–15.53)	45.30 (29.33–135.29)	<0.001 *
SIT (ng/dL)	NA	NA	5.51 (5.01–6.83)	20 (10.6–39.5)	0.002 *

Data are presented as median (interquartile range) or *n* (%). Comparisons were performed using the Kruskal–Wallis test for continuous variables and the chi-square test for categorical variables. Analyses were performed using complete-case data for each variable. SIT was performed in a limited number of patients, predominantly in the PA group. * *p* < 0.05 was considered statistically significant. Abbreviations: CS, Cushing’s syndrome; MACS, Mild autonomous cortisol secretion; NFA, non-functioning adenoma; PA, primary aldosteronism; BMI, body mass index; WC, waist circumference; HT, hypertension; DM, diabetes mellitus; PreDM, prediabetes; HDL-C, high-density lipoprotein cholesterol; LDL-C, low-density lipoprotein cholesterol; ACTH, adrenocorticotropic hormone; DHEAS, dehydroepiandrosterone sulfate; DST, dexamethasone suppression test; ARR, aldosterone-to-renin ratio; SIT, saline infusion test.

**Table 2:** Adjusted comparison of CT-derived parameters across adrenal adenoma subtypes.

Variable	CS (*n* = 31)	MACS (*n* = 35)	NFA (*n* = 61)	PA (*n* = 44)	*p*-Value
VFA (cm^2^)	112.89 ± 6.85	95.62 ± 6.29	113.13 ± 4.63	111.16 ± 5.43	0.133
SFA (cm^2^)	129.65 ± 7.47	121.87 ± 6.86	139.57 ± 5.05	121.39 ± 5.92	0.072
VFA/SFA ratio	0.86 ± 0.08	0.85 ± 0.07	0.85 ± 0.05	1.07 ± 0.06	0.039 *
Psoas muscle area (cm^2^)	11.98 ± 0.78	13.93 ± 0.71	13.12 ± 0.52	13.87 ± 0.61	0.221
Non-psoas skeletal muscle area (cm^2^)	71.77 ± 2.85	82.03 ± 2.62	88.34 ± 1.93	96.56 ± 2.26	<0.001 *
Total skeletal muscle area (cm^2^)	83.74 ± 3.26	95.96 ± 2.99	101.46 ± 2.20	110.42 ± 2.58	<0.001 *
PMI (cm^2^/m^2^)	4.35 ± 0.27	5.08 ± 0.25	4.91 ± 0.18	4.82 ± 0.21	0.247
Non-psoas SMI (cm^2^/m^2^)	26.42 ± 0.90	30.27 ± 0.83	33.38 ± 0.61	34.35 ± 0.71	<0.001 *
Total SMI (cm^2^/m^2^)	30.77 ± 1.01	35.35 ± 0.93	38.29 ± 0.68	39.17 ± 0.80	<0.001 *
Muscle attenuation (HU)	32.57 ± 0.70	35.42 ± 0.64	35.87 ± 0.47	37.52 ± 0.55	<0.001 *

Data are presented as estimated marginal means ± standard error. Comparisons were performed using analysis of covariance (ANCOVA), adjusted for age, sex, and body mass index (BMI). Analyses were performed using complete-case data for each variable. * *p* < 0.05 was considered statistically significant. Abbreviations: CS, Cushing’s syndrome; MACS, Mild autonomous cortisol secretion; NFA, non-functioning adenoma; PA, primary aldosteronism; VFA, visceral fat area; SFA, subcutaneous fat area; PMI, psoas muscle index; SMI, skeletal muscle index; HU, Hounsfield unit.

**Table 3:** Adjusted pairwise comparisons of CT-derived parameters across adrenal adenoma subtypes.

Variable	CS vs. MACS	CS vs. NFA	CS vs. PA	MACS vs. NFA	MACS vs. PA	NFA vs. PA
VFA	0.450	1.000	1.000	0.157	0.380	1.000
SFA	1.000	1.000	1.000	0.234	1.000	0.123
VFA/SFA ratio	1.000	1.000	0.249	1.000	0.157	0.060
Psoas muscle area	0.453	1.000	0.351	1.000	1.000	1.000
Non-psoas skeletal muscle area	0.069	<0.001 *	<0.001 *	0.323	<0.001 *	0.037 *
Total skeletal muscle area	0.051	<0.001 *	<0.001 *	0.838	0.002 *	0.054
PMI	0.327	0.512	1.000	1.000	1.000	1.000
Non-psoas SMI	0.017 *	<0.001 *	<0.001 *	0.017 *	0.002 *	1.000
Total SMI	0.009 *	<0.001 *	<0.001 *	0.069	0.013 *	1.000
Muscle attenuation	0.025 *	0.001 *	<0.001 *	1.000	0.088	0.147

Pairwise comparisons were performed using analysis of covariance (ANCOVA) with Bonferroni correction for multiple comparisons, adjusted for age, sex, and body mass index (BMI). Analyses were conducted using estimated marginal means. * *p* < 0.05 was considered statistically significant. Abbreviations: CS, Cushing’s syndrome; MACS, Mild autonomous cortisol secretion; NFA, non-functioning adenoma; PA, primary aldosteronism; PMI, psoas muscle index; SMI, skeletal muscle index.

**Table 4:** Hormone-specific correlations with CT-derived body composition parameters.

Variable	1 mg DST Cortisol		Aldosterone		ARR	
	r	*p* Value	r	*p* Value	r	*p* Value
VFA	0.072	0.445	0.154	0.196	0.101	0.399
SFA	0.026	0.781	−0.061	0.612	−0.150	0.207
VFA/SFA ratio	−0.005	0.957	0.308	0.009 *	0.272	0.021 *
Psoas muscle area	−0.237	0.011 *	0.197	0.097	0.185	0.121
Non-psoas skeletal muscle area	−0.554	<0.001 *	0.152	0.202	0.239	0.043 *
Total skeletal muscle area	−0.519	<0.001 *	0.184	0.121	0.240	0.042 *
PMI	−0.229	0.014 *	0.196	0.099	0.112	0.348
Non-psoas SMI	−0.496	<0.001 *	0.124	0.299	0.107	0.371
Total SMI	−0.484	<0.001 *	0.146	0.221	0.100	0.405
Muscle attenuation	−0.384	<0.001 *	0.169	0.155	0.154	0.198

Spearman correlation analysis was performed to assess the associations between hormonal parameters and CT-derived body composition measures. Correlations were assessed using 1 mg DST values in patients with CS, MACS, and NFA and using renin–aldosterone system parameters (aldosterone and ARR) in patients with PA and NFA, based on available data. Analyses were conducted using available data for each variable. * *p* < 0.05 was considered statistically significant. Abbreviations: DST, dexamethasone suppression test; ARR, aldosterone-to-renin ratio; VFA, visceral fat area; SFA, subcutaneous fat area; PMI, psoas muscle index; SMI, skeletal muscle index.

**Table 5:** Multivariable associations between 1 mg DST cortisol and CT-derived skeletal muscle parameters.

Variable	Model 1		Model 2	
	β	*p* Value	β	*p* Value
Psoas muscle area	−0.186	0.009 *	−0.107	0.310
Non-psoas skeletal muscle area	−0.340	<0.001 *	−0.214	0.019 *
Total skeletal muscle area	−0.332	<0.001 *	−0.205	0.041 *
PMI	−0.210	0.009 *	−0.121	0.220
Non-psoas SMI	−0.396	<0.001 *	−0.251	0.011 *
Total SMI	−0.339	<0.001 *	−0.209	0.034 *
Muscle attenuation	−0.301	0.004 *	−0.291	0.019 *

Multivariable linear regression analysis was performed to assess the associations between 1 mg DST cortisol levels and CT-derived skeletal muscle parameters. Model 1 was adjusted for age, sex, and BMI. Model 2 was additionally adjusted for hormonal subgroup (CS, MACS, and NFA). Patients with PA were not included in these analyses. β values represent standardized regression coefficients. * *p* < 0.05 was considered statistically significant. Abbreviations: DST, dexamethasone suppression test; BMI, body mass index; CS, Cushing syndrome; MACS, mild autonomous cortisol secretion; NFA, non-functioning adenoma; PA, primary aldosteronism; PMI, psoas muscle index; SMI, skeletal muscle index.

## Data Availability

The data presented in this study are available from the corresponding author upon reasonable request. The data are not publicly available because they contain information that could compromise the privacy of research participants.

## Supplementary Materials

The following supporting information can be downloaded at: https://www.mdpi.com/article/10.3390/jcm15176861/s1, Supplementary Table S1. Interobserver agreement for CT-derived imaging measurements. Supplementary Table S2. CT-based body composition parameters according to adrenal adenoma subtype.

## References

[1] Ebbehoj A., Li D., Kaur R.J., Zhang C., Singh S., Li T., Atkinson E., Achenbach S., Khosla S., Arlt W. (2020). Epidemiology of adrenal tumours in Olmsted County, Minnesota, USA: A population-based cohort study. Lancet Diabetes Endocrinol..

[2] Barzon L., Sonino N., Fallo F., Palu G., Boscaro M. (2003). Prevalence and natural history of adrenal incidentalomas. Eur. J. Endocrinol..

[3] Mansmann G., Lau J., Balk E., Rothberg M., Miyachi Y., Bornstein S.R. (2004). The clinically inapparent adrenal mass: Update in diagnosis and management. Endocr. Rev..

[4] Bancos I., Prete A. (2021). Approach to the Patient with Adrenal Incidentaloma. J. Clin. Endocrinol. Metab..

[5] Iñiguez-Ariza N.M., Kohlenberg J.D., Delivanis D.A., Hartman R.P., Dean D.S., Thomas M.A., Shah M.Z., Herndon J., McKenzie T.J., Arlt W. (2018). Clinical, Biochemical, and Radiological Characteristics of a Single-Center Retrospective Cohort of 705 Large Adrenal Tumors. Mayo Clin. Proc. Innov. Qual. Outcomes.

[6] Ichijo T., Ueshiba H., Nawata H., Yanase T. (2020). A nationwide survey of adrenal incidentalomas in Japan: The first report of clinical and epidemiological features. Endocr. J..

[7] Lee Y.S., Hong N., Witanto J.N., Choi Y.R., Park J., Decazes P., Eude F., Kim C.O., Kim H.C., Goo J.M. (2021). Deep neural network for automatic volumetric segmentation of whole-body CT images for body composition assessment. Clin. Nutr..

[8] Kim J.H., Jang H.N., Park S.S., Yoon J.H., Cho Y.M., Park S.J., Lee J.M., Yoon J.W. (2025). Body composition and cardiometabolic risks of patients with adrenal tumours in relation to hormonal activity: A large cross-sectional single-centre study. Eur. J. Endocrinol..

[9] Liu D., Garrett J.W., Lee M.H., Zea R., Summers R.M., Pickhardt P.J. (2023). Fully automated CT-based adiposity assessment: Comparison of the L1 and L3 vertebral levels for opportunistic prediction. Abdom. Radiol..

[10] Hamaguchi Y., Kaido T., Okumura S., Kobayashi A., Hammad A., Tamai Y., Inagaki N., Uemoto S. (2016). Proposal for new diagnostic criteria for low skeletal muscle mass based on computed tomography imaging in Asian adults. Nutrition.

[11] Uchida T., Yamaguchi H., Arimura Y., Nagayama A., Moritaka K., Inoguchi Y., Ashida K., Nomura M., Nakazato M., Shimoda K. (2023). Iliopsoas muscle to visceral fat ratio on CT predicts Cushing’s syndrome in elderly females with adrenal tumors. Endocr. J..

[12] Hong N., Lee J., Ku C.R., Han K., Lee C.R., Kang S.W., Rhee Y. (2019). Changes of computed tomography-based body composition after adrenalectomy in patients with endogenous hypercortisolism. Clin. Endocrinol..

[13] Delivanis D.A., Hurtado Andrade M.D., Cortes T., Athimulam S., Khanna A., Atkinson E., McKenzie T., Takahashi N., Moynagh M.R., Bancos I. (2021). Abnormal body composition in patients with adrenal adenomas. Eur. J. Endocrinol..

[14] Chen K.M., Lee B.C., Chen P.T., Liu K.L., Lin K.H., Chang C.C., Wu T.H., Hong J.S., Lin Y.H. (2021). Evaluation of Abdominal Computed Tomography Scans for Differentiating the Discrepancies in Abdominal Adipose Tissue Between Two Major Subtypes of Primary Aldosteronism. Front. Endocrinol..

[15] Ko Y., Jeong H., Khang S., Lee J., Kim K.W., Kim B.J. (2022). Change of Computed Tomography-Based Body Composition after Adrenalectomy in Patients with Pheochromocytoma. Cancers.

[16] Delivanis D.A., Iñiguez-Ariza N.M., Zeb M.H., Moynagh M.R., Takahashi N., McKenzie T.J., Thomas M.A., Gogos C., Young W.F., Bancos I. (2018). Impact of hypercortisolism on skeletal muscle mass and adipose tissue mass in patients with adrenal adenomas. Clin. Endocrinol..

[17] Schakman O., Kalista S., Barbé C., Loumaye A., Thissen J.P. (2013). Glucocorticoid-induced skeletal muscle atrophy. Int. J. Biochem. Cell Biol..

[18] Arbanas J., Klasan G.S., Nikolic M., Jerkovic R., Miljanovic I., Malnar D. (2009). Fibre type composition of the human psoas major muscle with regard to the level of its origin. J. Anat..

[19] Lee B.C., Chang Y.L., Chen P.T., Liu L.W., Liu K.L., Chang C.C., Wu V.C., Lin Y.H. (2025). Myosteatosis and sarcopenia are linked to autonomous cortisol secretion in patients with aldosterone-producing adenomas. Hypertens. Res. Off. J. Jpn. Soc. Hypertens..

