# Supplementary material for: Association of Hormonal Activity with CT-Derived Body Composition Characteristics in Patients with Adrenal Adenomas
Source: J Clin Med. 2026 Sep 4;15(17):6861. doi: 10.3390/jcm15176861 (PMC13566672; doi:10.3390/jcm15176861)
Supplement: Supplementary file 1 [file jcm-15-06861-s001.zip › jcm-4480210-supplementary.pdf]

Supplementary Table S1. Interobserver agreement for CT-derived imaging measurements.

Parameter	ICC	95% CI
**Tumor size**	0.956	0.914–0.977
**Psoas muscle area**	0.921	0.899–0.941
**VFA**	0.918	0.890–0.943
**SFA**	0.911	0.887–0.928
**Non-psoas skeletal muscle area**	0.932	0.907–0.961
**Muscle attenuation**	0.942	0.907–0.975

Interobserver agreement was assessed using a two-way random-effects model for absolute agreement based on single measurements. ICC values are presented with 95% confidence intervals (CI).

Abbreviations: ICC, intraclass correlation coefficient; CI, confidence interval; CT, computed tomography; VFA, visceral fat area; SFA, subcutaneous fat area.

Supplementary Table S2. CT-based body composition parameters according to adrenal adenoma subtype.

Variable	**CS** (n=31)	MACS (n=35)	**NFA** (n=61)	**PA** (n=44)	p-value
**Tumor size (mm)**	32 (27–37)	41 (25–57)	35 (27–43)	18.5 (15–24)	**<0.001***
**VFA (cm^2)**	128 (83–166)	104 (78–129)	113 (82–153)	112.5 (82–134)	0.366
**SFA (cm^2)**	143 (81–162)	120 (76–165)	142 (83–162)	122.5 (76–150)	0.302
**VFA/SFA ratio**	0.89 (0.73–1.02)	0.85 (0.68–0.99)	0.87 (0.70–1.00)	0.92 (0.70–1.13)	0.356
**Psoas muscle area (cm^2)**	10.1 (7.8–12.8)	13.3 (10.7–18.3)	12.6 (9.8–15.9)	13.0 (9.0–18.0)	**0.006***
**Non-psoas skeletal muscle area (cm^2)**	68 (60–75)	82 (72–90)	88 (81–96)	88.5 (76–103)	**<0.001***
**Total skeletal muscle area (cm^2)**	78.1 (72–92)	92.7 (84–112)	102.6 (92–112)	100.9 (84–127)	**<0.001***
**PMI (cm^2/m^2)**	3.95 (3.2–5.0)	4.61 (3.7–6.1)	4.87 (3.8–6.0)	4.69 (3.7–6.0)	**0.014***
**Non-psoas SMI (cm^2/m^2)**	27.1 (24–30)	29.4 (26–34)	32.9 (30–36)	33.1 (28–38)	**<0.001***
**Total SMI (cm^2/m^2)**	31.0 (28–34)	36.2 (30–40)	38.4 (35–41)	37.3 (31–43)	**<0.001***
**Muscle attenuation (HU)**	33 (30–35)	36 (33–38)	36 (33–39)	38 (34–41)	**<0.001***

Data are presented as median (interquartile range).
Comparisons were performed using the Kruskal–Wallis test.

Analyses were performed using complete-case data for each variable.
* $p < 0.05$ was considered statistically significant.

Abbreviations: CS, Cushing's syndrome; MACS, Mild autonomous cortisol secretion; NFA, non-functioning adenoma; PA, primary aldosteronism; VFA, visceral fat area; SFA, subcutaneous fat area; PMI, psoas muscle index; SMI, skeletal muscle index; HU, Hounsfield unit
